# Identification of CCL19 as a Novel Immune-Related Biomarker in Diabetic Nephropathy

**DOI:** 10.3389/fgene.2022.830437

**Published:** 2022-02-09

**Authors:** Hanzhi Chen, Zhijian Zhang, Leting Zhou, Ting Cai, Bin Liu, Liang Wang, Junwei Yang

**Affiliations:** ^1^ Center for Kidney Disease, The Second Affiliated Hospital of Nanjing Medical University, Nanjing, China; ^2^ Department of Nephrology, The Affiliated Wuxi People’s Hospital of Nanjing Medical University, Wuxi, China

**Keywords:** diabetic nephropathy, immune cell, CCL19, bioinformatics, biomarker

## Abstract

Diabetic nephropathy (DN) is one of the major microvascular complications in diabetic patients and the leading cause of end-stage renal disease (ESRD). Previous studies found that immune-related genes and immune cell infiltration play important roles in the pathogenesis and development of DN. Therefore, this study aimed to explore immune-related biomarkers in DN. In this research, three microarray datasets that included 18 DN and 28 healthy tubule samples were downloaded and integrated as the training set to identify differentially expressed immune-related genes (DEIGs). A total of 63 DEIGs were identified, and most upregulated DEIGs were primarily involved in the inflammatory response and chemokine-mediated signaling pathways. The Microenvironment Cell Populations-counter (MCP-counter) algorithm was then used to estimate the abundance of infiltrated immune and stromal cell populations. According to DEIG, weighted gene coexpression network and protein–protein network analyses, CCL19 was identified as the hub immune-related biomarker. Moreover, the upregulated level of CCL19 was confirmed in other independent datasets as well as in *in vitro* experiments with high glucose. In summary, this study provides novel insights into the pathogenesis of diabetic nephropathy and identifies CCL19 as a potential critical gene of DN.

## Introduction

Diabetic nephropathy (DN) is one of the major microvascular complications in diabetic patients and the leading cause of end-stage renal disease (ESRD) (Lu et al., 2017). It is generally acknowledged that inhibitors of the renin/angiotensin axis are a widely used therapeutic drug for DN that downregulates the levels of both blood pressure and blood glucose (Rahimi, 2016). Because the pathogenesis of DN is not completely understood, effective therapeutic drugs are still limited. According to authoritative statistics, the number of deaths caused by DN increased by more than 90% from 1990 to 2012 (Doshi and Friedman, 2017). In the preceding decades, an increasing number of studies contributed to the pathogenesis of DN from the perspective of molecular mechanisms, and the pathogenic roles of signaling pathway abnormalities have been preliminarily recognized (Brosius et al., 2010; Gu et al., 2020; Zhou et al., 2020). However, due to the complexity of the pathogenesis of DN, additional molecular mechanisms related to the etiology of DN need to be further explored.

Previous studies reported that metabolic and hemodynamic factors are the major causes of DN (Kanwar et al., 2011). However, increasing evidence reveals that inflammation and tissue-infiltrated immune cell populations contribute to the pathogenesis and progression of DN (Tang and Yiu, 2020). Compared with healthy controls, large amounts of inflammatory cells infiltrate tubules in patients with DN (Klessens et al., 2017), and inflammatory factors, such as interleukin (IL)-16 and IL-18, are also increased in the serum or peripheral blood of patients with DN (Perlman et al., 2015). Thus, exploring the immune mechanisms of DN provides new insights into the pathogenesis and development of DN.

In the current study, we utilized public datasets of DN and healthy tubule samples from the Gene Expression Omnibus (GEO) database to explore potential immune-related biomarkers in DN by performing bioinformatics approaches. Our results showed that chemokine (C-C motif) ligand 19 (CCL19) was upregulated significantly in the DN samples and displayed excellent diagnostic value for DN and healthy samples. Furthermore, the transcriptional and protein levels of CCL19 were also upregulated in HK-2 cells under high glucose stimulation. Overall, these data reveal that CCL19 is a potential critical gene in the pathogenesis of DN.

## Materials and Methods

### Acquirement and Analysis of Gene Expression Omnibus Datasets

The GEO portal was used to obtain gene expression datasets from human tubules of DN patients and healthy controls. The screening criteria included the following: 1) *Homo sapiens* gene expression profiling by array or high-throughput sequencing; 2) tubules of DN patients or healthy controls; and 3) datasets containing more than five samples. Finally, five DN-related datasets, GSE104954, GSE30529, GSE35487, GSE37463 and GSE47184, were obtained. Three datasets (GSE35487, GSE37463, and GSE47184), which included 18 DN samples and 28 healthy control samples, were merged as the training cohort, and another two datasets (GSE104954 and GSE30529) were used as the test cohort. The robust multiarray average (RMA) algorithm in the affy package was performed to preprocess the array profiles. After background correction, quantile normalization and probe summarization, the gene expression profile was obtained according to the gene and probe mapping platform. The batch effects were eliminated using the function “removeBatchEffect” in the R package limma. Moreover, the distribution patterns in the datasets and DN and healthy samples after eliminating batch effects were evaluated using a two-dimensional principal component analysis (PCA). All details for these datasets are presented in Table 1.

**TABLE 1:** The information of GEO datasets in this study.

GEO series	DN	Normal	Tissue	Data type	Gene number
GSE104954	17	21	tubules	test	11720
GSE30529	10	12	tubules	test	12548
GSE35487	0	6	tubules	train	12548
GSE37463	0	18	tubules	train	11959
GSE47184	18	4	tubules	train	11959

### Screening of Differentially Expressed Immune-Related Genes

First, we collected 3,472 immune-related genes from InnateDB (Breuer et al., 2013), TISIDB (Ru et al., 2019), and Immport (Bhattacharya et al., 2018), which are comprehensive datasets that curate immune-related genes from research articles. Second, we intersected these immune-related genes in the training cohort and acquired the gene expression profile with 2,269 immune-related genes. A differential expression analysis was performed between the DN and healthy groups using the R package limma. Then, immune-related genes were identified as DEIGs at a |log-fold change (FC)| ≥ 1 and Benjamini and Hochberg adjusted *p* value ≤ 0.05.

### Enrichment Analysis of Gene Functions and Pathways

A Gene Ontology (GO) pathway enrichment analysis for DEIGs upregulated in the DN samples was performed using the Enrichr database (Kuleshov et al., 2016). The top 10 enriched GO pathways were displayed on the webpage and downloaded as images.

### Evaluation of Tissue-Infiltrating Immune and Stromal Cell Populations

Considering that the bulk transcriptomic data from the DN and healthy samples included both immune and stromal cell populations, we used the Microenvironment Cell Populations-counter (MCP-counter) method (Becht et al., 2016), which performs robust quantifications of the absolute abundance of eight immune and two stromal cell populations based on the methodological framework (Shen-Orr and Gaujoux, 2013) of transcriptomic markers, to estimate the population abundance of tissue-infiltrating immune and stromal cells in the training cohort. A PCA was performed to explore the distribution patterns of the DN and healthy samples in training cohorts based on the population abundance calculated by MCP-counter. The population abundance of infiltrated immune and stromal cells in the DN and healthy groups was visualized in boxplots. We then selected the significant immune cell populations between the DN and healthy groups for further analysis.

### Identification of Significant Modules With Immune Infiltration Characteristics

To further understand the association between gene expression and immune cell population abundance, we constructed a weighted coexpression network (WGCNA) according to the expression profile of the top 25% variable genes (2,715 genes) by utilizing the R package WGCNA (Langfelder and Horvath, 2008) and then identified the significant gene modules related to the infiltrated immune cells.

When applying a soft threshold, the elements in the adjacency matrix are continually elementized through a weight function. Because the choice of the soft threshold (β) will likely affect the module identification results and the relative network of the random average of each node, a scale-free network is implemented in which a few nodes exhibit a significantly higher degree than the general point, which is a more stable choice. Therefore, the soft threshold (β) for our gene distribution must be consistent with the scale-free network. To create a network with a nearly scale-free topology, a soft threshold *R* ^2^ of 0.89 (*β* = 14) was set for further analysis. Pearson’s correlation test was used to assess the correlation between the abundance of infiltrated immune cells and modules and identify the meaningful modules. Module membership (MM) and gene significance (GS) revealed the correlation between the gene expression and infiltrated immune cell abundance. Genes in modules that were highly associated with infiltrated immune cells (correlation ≥ 0.7 and *p* value ≤ 0.05) were extracted for further study (GS ≥ 0.5 and MM ≥ 0.5). Candidate biomarkers were identified by cross-linking the genes obtained from the WGCNA and DEIG analysis.

### Protein–Protein Interaction Network Construction

The PPI network was constructed using the STRING database (https://string‐db.org/) to demonstrate the functional interactions among proteins. The intersecting genes from the WGCNA and DEIG analyses were used to construct the PPT network. Then, we identified the hub gene CCL19 by maximal clique centrality (MCC), a newly proposed method with better performance in predicting essential proteins from the PPI network (Chin et al., 2014), using Cytoscape software (version 3.7.2) (Chin et al., 2014). The receiver operating curve (ROC) was plotted using the R package pROC to estimate the capability of CCL19 to distinguish DN patients from healthy controls.

### Human Protein Atlas Database Analysis

The Human Protein Atlas (HPA, http://www.proteinatlas.org/) was launched to depict the landscape of human proteins using the integration of multiomics (Uhlen et al., 2010; Uhlén et al., 2015). The HPA platform allows all researchers to freely acquire data to study the human proteome. In this study, the levels of CCL19 protein expression in human tissues were explored using the HPA dataset. The HPA dataset also offers single-cell RNA-sequencing (scRNA-seq) data, and we also investigated the landscape of CCL19 in various cell types in kidney tissues using the HPA dataset.

### Cell Culture

HK-2 cells, a renal tubular epithelial cell line, were obtained from KeyGEN BioTECH (Nanjing, China). HK-2 cells were cultured in DMEM medium (Cat. 11966–025, Gibco) and F12 medium (Cat. 11765–054, Gibco) (1:1 allocation) supplemented with 10% fetal bovine serum (Thermo Scientific) at 37 °C with 5% CO_2_. For the subsequent assay, HK-2 cells were cultured in a high glucose (29.63 mmol/L) environment. Next, control and experimental cells were submitted for quantitative real-time PCR (qRT–PCR) and western blotting analyses.

### Total RNA Extraction and qRT–PCR

Total RNA was extracted from HK-2 cells using TRIzol solution (Invitrogen), and the RNA specimens were stored in a −80°C freezer. Reverse transcription was conducted using PrimeScript RT reagent (TaKaRa) according to the manufacturer’s instructions. PCR was conducted using SYBR Green Master Mix II (TaKaRa). Each sample was repeated three times. The 2^−ΔΔCt^ method was applied for CCL19 expression analysis, with the expression levels normalized to that of GAPDH. Specific gene primer sequences were as follows: CCL19: 5′-CCA​ACT​CTG​AGT​GGC​ACC​AA-3′ (forward), 5′-TGA​ACA​CTA​CAG​CAG​GCA​CC-3′ (reverse); GAPDH: 5′-AGA​TCA​TCA​GCA​ATG​CCT​CCT-3′ (forward), 5′- TGA​GTC​CTT​CCA​CGA​TAC​CAA -3′ (reverse).

### Protein Extraction and Western Blotting Analysis

For protein extraction, HK-2 cells were washed three times with PBS, and then the proteins were harvested with lysis buffer. SDS-polyacrylamide gel electrophoresis and western blotting analysis were conducted by standard protocols. The primary antibodies used were as follows: anti-CCL19 (1:1,000 dilution, Cat. ab192871, Abcam) and β-actin (1:5,000 dilution, Cat. 60008-1-Ig, ProteinTech). The protein expression level of CCL19 was normalized to that of β-actin for each sample.

### Statistical Analysis

All statistical analyses were performed using R-4.0.4. The Wilcoxon rank-sum test was performed to compare the differences between the DN and healthy groups. Differences were considered statistically significant at *p* ≤ 0.05.

## Results

### Differentially Expressed Immune-Related Genes Identification Between the DN and Healthy Tubule Samples and Functional Analysis

**FIGURE 1:**
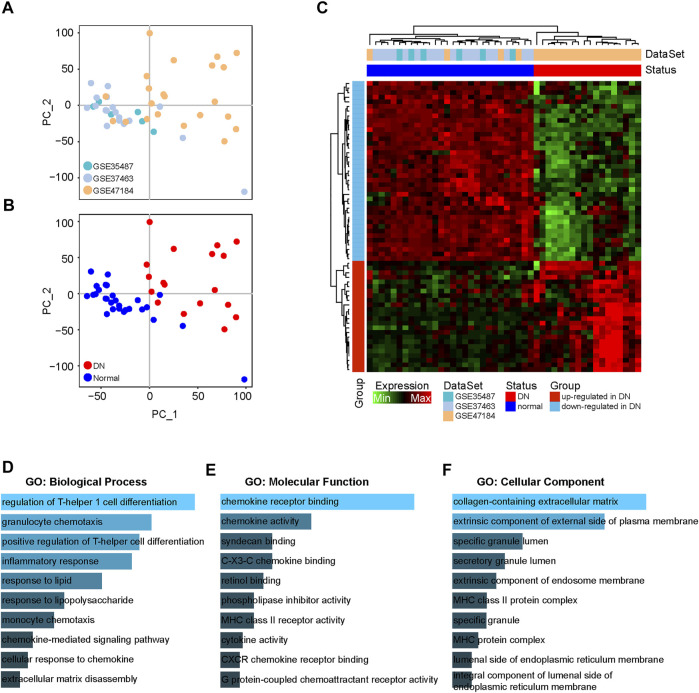
Differential expression analysis of immune-related genes between DN and healthy samples. **(A)** PCA figures displaying interbatch differences removed after integrating GSE35487, GSE37463 and GSE47184. **(B)** PCA figures displaying batch differences for the DN and healthy samples after integrating GSE35487, GSE37463 and GSE47184. **(C)** Heatmap showing the DEIGs between the DN and healthy samples. **(D–F)** GO analyses of DEIGs in terms of biological process, molecular function and cellular components.

To explore the immune-related genes participating in the pathogenesis of DN, we collected a total of 18 DN and 28 healthy tubule samples from GSE35487, GSE37463 and GSE47184. The interbatch differences were removed from the gene expression profile after integrating these datasets. The PCA results showed the distribution of the datasets and DN and healthy samples after removing the batch effects (Figures 1A,B), and they indicated that the batch effects between the different datasets were eliminated. Next, we performed a differential expression analysis to screen the DEIGs between the DN and healthy samples based on the integrated gene expression profile (Supplementary Table S1). With |logFC| ≥ 1 and adjusted *p* value ≤ 0.05, a total of 63 DEIGs were identified, including 39 downregulated and 24 upregulated immune-related genes in the DN samples compared to the healthy samples (Figure 1C).

To further explore the biological functions of the downregulated and upregulated DEIGs in the DN samples, a functional enrichment analysis was performed in the Enrichr database. The GO analysis identified major enrichment in the biological processes category, including in the inflammatory response and chemokine-mediated signaling pathways (Figure 1D). In addition, in the molecular function category, several main pathways were associated with chemokines, including chemokine and cytokine activity (Figure 1E). Moreover, primary enrichment in the cellular components category occurred for specific and secretory granule lumens (Figure 1F).

### Tissue-Infiltrated Immune and Stromal Cell Population Analysis

**FIGURE 2:**
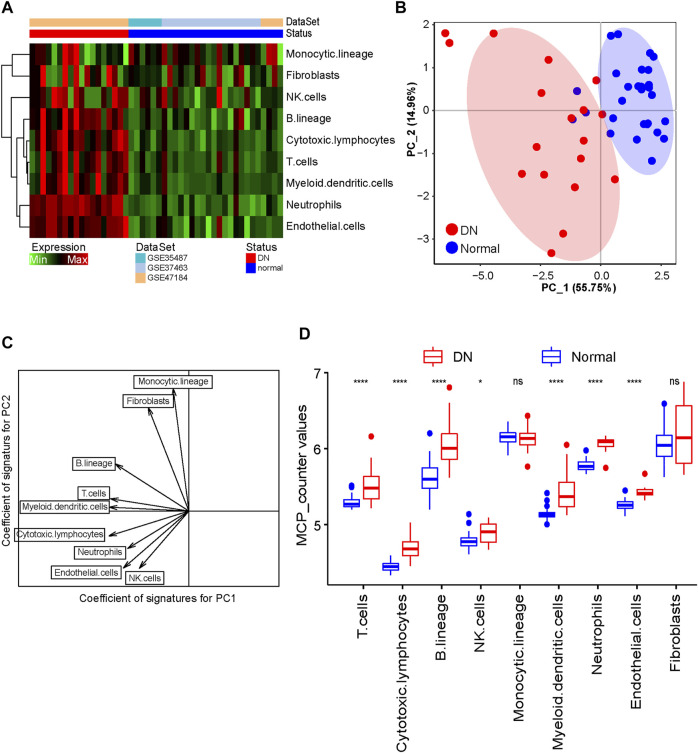
Evaluation of tissue-infiltrating immune and stromal cell populations. **(A)** Heatmap showing the abundance of tissue-infiltrated immune and stromal cell populations between DN and healthy samples. **(B)** PCA figure displaying the distribution of DN and healthy samples. **(C)** Scatter diagram of the vector basis projection showing the contribution degree of cell populations. The length of the arrow indicates the absolute contribution degree. **(D)** Boxplot showing the abundance of infiltrated immune cell populations in DN and healthy samples. The Wilcoxon rank-sum test was used to measure the differences between two groups. Ns, no significant difference; ^*^ *p* < 0.05; ^****^ *p* < 0.0001.

Given the limitations of technology, the tissue-infiltrated immune and stromal cell populations of the DN and healthy tubule samples were not fully elucidated. By utilizing the MCP-counter algorithm, we estimated the population abundance of tissue-infiltrated immune and stromal cells between the DN and healthy samples according to the gene expression profile of the training cohort and displayed the results in Figure 2A. The PCA showed that the population abundance of infiltrated immune and stromal cells of the healthy samples was greatly different from that of the DN samples (Figure 2B). Specifically, the DN and normal samples can be distinguished according to PC1, in which cytotoxic lymphocytes and myeloid dendritic cells had the highest absolute contribution degree. In addition, monocytic lineage and fibroblasts showed the lowest absolute contribution degree for PC1 but the highest absolute coefficients for PC2 (Figure 2C; Supplementary Table S2). Further analysis showed that the abundance of almost all immune cell populations in the DN samples was significantly higher than that in the healthy samples, especially cytotoxic lymphocytes and myeloid dendritic cells, indicating that infiltrated immune cells play an important role in the pathogenesis of DN (Figure 2D).

### Identification of Immune-Related Gene Modules

**FIGURE 3:**
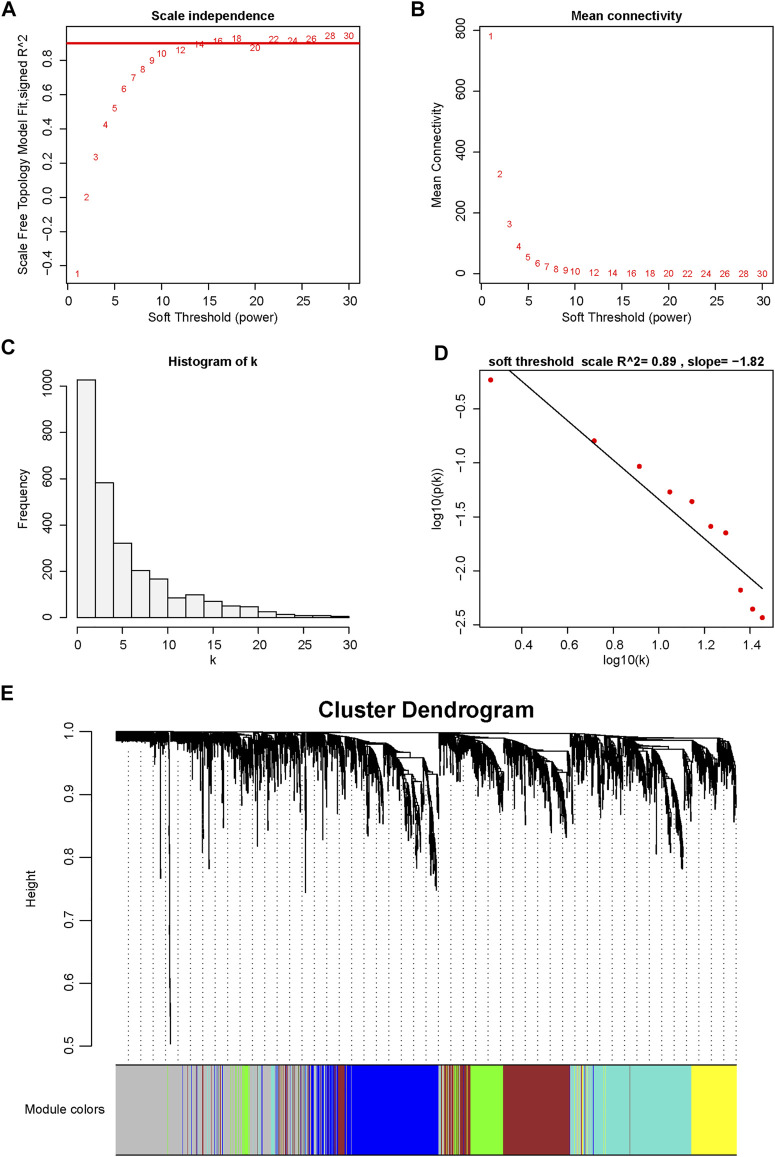
Determination of soft-thresholding power in WGCNA. **(A)** Analysis of the scale-free fitting indices for various soft-thresholding powers (β). **(B)** Mean connectivity analysis of various soft-thresholding powers. **(C)** Histogram of the connection distribution when *β* = 12. **(D)** Checking the scale-free topology when *β* = 12. **(E)** Cluster dendrogram of the coexpression network modules is ordered by hierarchical clustering of genes based on the 1-TOX matrix. Different colors represent different modules.

**FIGURE 4:**
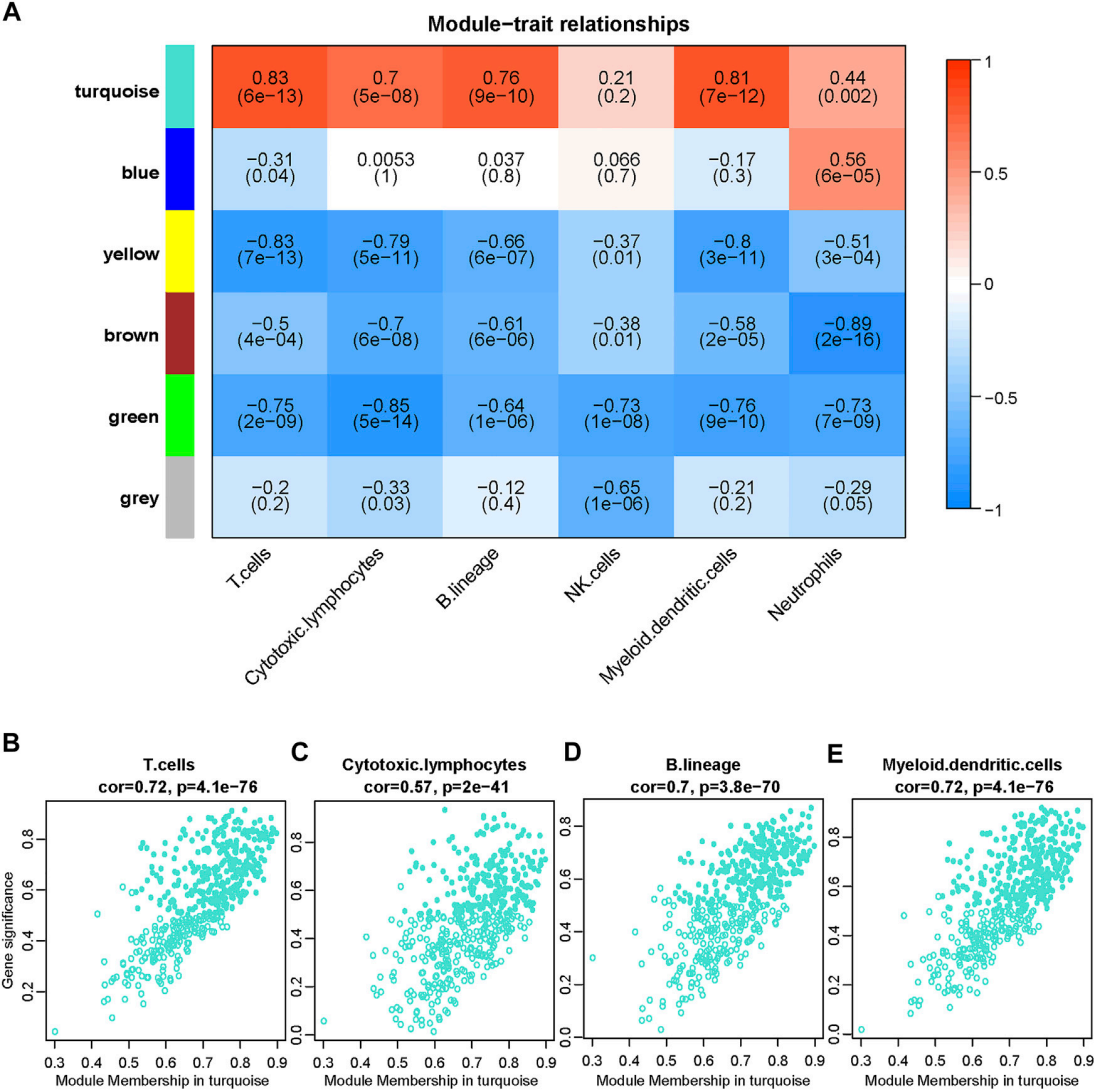
Screening candidate immune-related DN biomarkers using WGCNA. **(A)** Correlation between the gene module and infiltrated immune cell population, which significantly differed between the DN and healthy samples. The correlation coefficient in each cell represented the correlation between the gene module and the infiltrated immune cell, which decreased in color from red to blue. The corresponding *p* value is also annotated. **(B–E)** Scatter plots depict the relationship between module membership (MM) and gene significance (GS) in the turquoise module. Genes with MM ≥ 0.5 and GS ≥ 0.5 are labeled with solid dots.

To further understand the association between gene expression and immune cell population abundance, we constructed a weighted coexpression network (WGCNA) using the R package WGCNA. The top 25% variable genes (2,715 genes) were included in the WGCNA. In our research, we selected the power of *β* = 12 (scale-free network *R* ^2^ = 0.89) as the soft threshold to ensure a scale-free network (Figures 3A–D). Then, six color-coded gene modules except the gray module, which included unclassified genes, were extracted for further analysis (Figure 3E). Figure 4A shows the correlation factors between gene modules and the abundance of infiltrated immune cell populations that were significantly enriched in the DN samples. The turquoise module had the highest correlation with the T cell (*R* = 0.83, *p* < 0.001), cytotoxic lymphocyte (*R* = 0.7, *p* < 0.001), B lineage (*R*= 0.76, *p* < 0.001) and myeloid dendritic cell (*R* = 0.81, *p* < 0.001) traits. The gene significance (GS) and module membership (MM) values for the turquoise module in the four immune cell populations are displayed in scatter plots, and genes with MM ≥ 0.5 and GS ≥ 0.5 were selected as candidate genes (Figures 4B–E).

### Identification and Validation of Hub Genes

**FIGURE 5:**
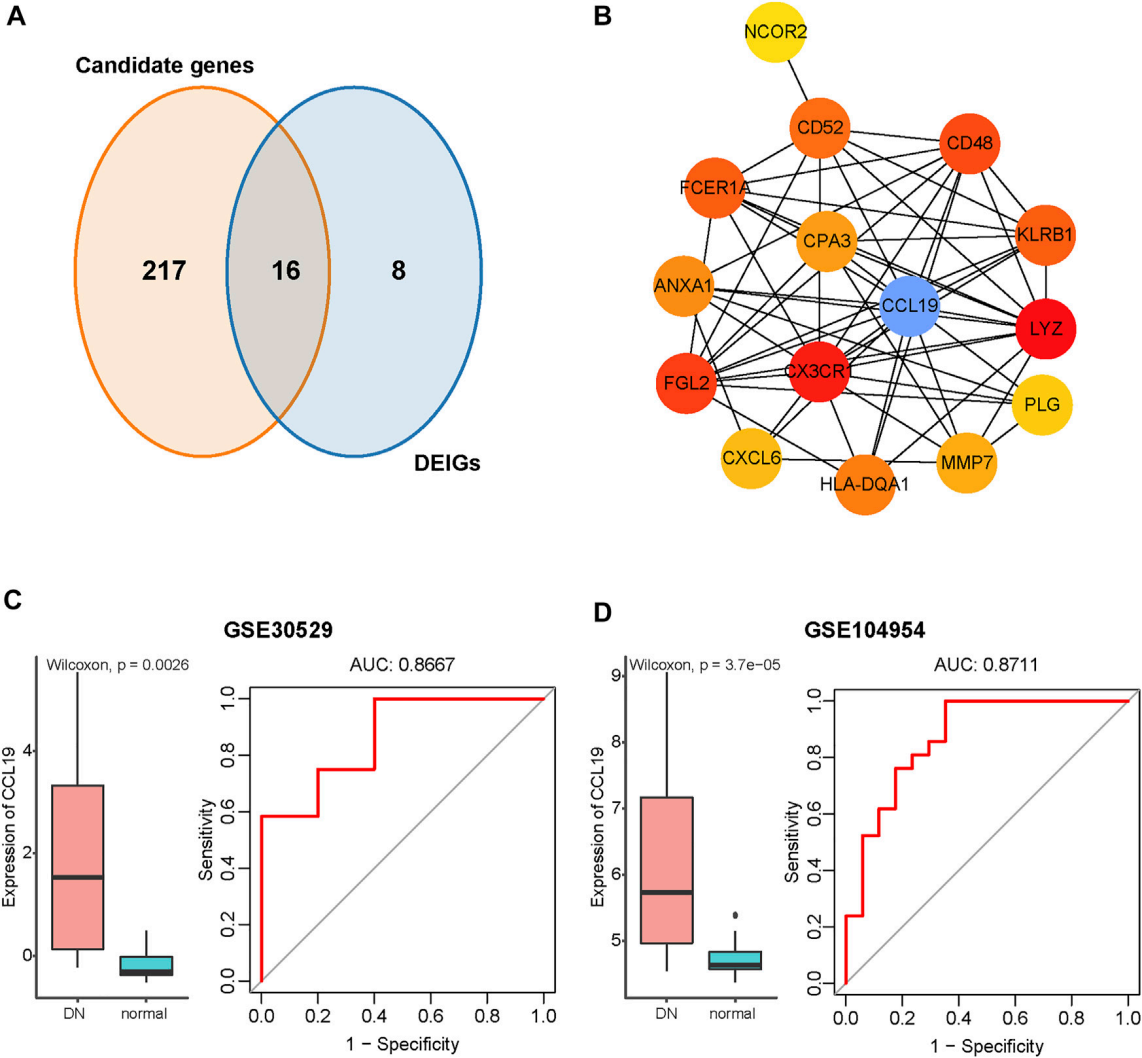
Identification of CCL19 as a hub immune gene in DN and verification in the GSE30529 and GSE104954 datasets. **(A)** Venn diagram of DEGs and immune-related gene list. **(B)** PPI network of the overlapping genes between WGCNA and DEIG analysis. The gradients from yellow to red of each circle represent the increasing degree of centrality. The blue circle represents the hub with the highest degree of centrality. **(C)** Verification of CCL19 in GSE30529. Left: Boxplot showing the expression of CCL19 between the DN and healthy samples in GSE30529. The Wilcoxon rank-sum test was used to measure the differences between two groups. Right: ROC curve of CCL19 expression in DN. **(D)** Verification of CCL19 in GSE104954. Left: Boxplot showing the expression of CCL19 between the DN and healthy samples in GSE104954. The Wilcoxon rank-sum test was used to measure the differences between two groups. Right: ROC curve of CCL19 expression in DN.

Sixteen overlapping genes among the DEIGs and candidate genes identified by the WGCNA were obtained for further analysis (Figure 5A). The protein–protein interaction (PPI) network of these genes was constructed using STRING. By applying the maximal clique centrality (MCC) algorithm, CCL19 was identified as a hub immune gene in DN (Figure 5B). To further validate the function of CCL19, we utilized another two independent datasets. The results showed that the expression of CCL19 increased significantly in the DN samples (GSE30529, *p* = 0.0026; GSE104954, *p* < 0.001; Figures 5C,D). Furthermore, the ROC analysis revealed that the expression values of CCL19 showed excellent diagnostic value for the DN and healthy samples (GSE305029, AUC = 0.87; GSE104954, AUC = 0.87; Figures 5C,D), indicating the potential role of CCL19 in the pathogenesis of DN.

### Validation of CCL19 Expression Status Under High Glucose Stimulation

**FIGURE 6:**
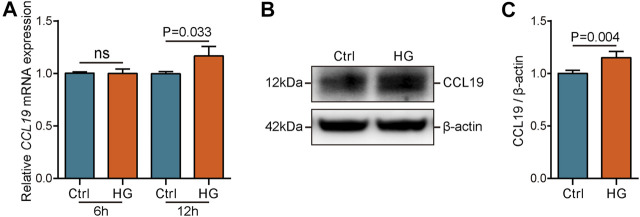
Validation of CCL19 using *in vivo* experiment. **(A)** mRNA level of CCL19 under high glucose stimulation was analyzed by qPCR. **(B,C)** Protein level of CCL19 under high glucose stimulation was analyzed by western blotting.

After identifying significantly the increased expression and excellent diagnostic value of CCL19 in the DN samples, we next explored the transcriptional and protein levels of CCL19 in healthy and HK-2 cells under stimulation with high glucose. First, we compared the expression values of CCL19 among healthy human tissues. The results showed that CCL19 was expressed at low levels in human tissues and was not expressed in human kidney tissues in the case of the healthy controls (Figure S1A-C). Then, we compared the transcriptional and protein levels of CCL19 between healthy and HK-2 cells after six or 12 hours of stimulation with high glucose. The results showed that the mRNA level of CCL19 was prominently upregulated after 12 h of stimulation with high glucose (Figure 6A). Furthermore, the protein level of CCL19 was also enhanced after 12 h of stimulation with high glucose (Figures 6B,C).

## Discussion

DN is a chronic inflammatory disease that causes podocyte injury, albuminuria, and loss of renal function (Wada and Makino, 2016). Recent studies found that immunomodulatory cytokines (Sabapathy et al., 2019) and immune cells (Fu et al., 2019) play critical roles in the pathogenesis of DN (Gurley et al., 2018). Therefore, further exploring the components of tissue-infiltrating immune cell populations in the DN samples and identifying immune-related biomarkers have great significance for understanding the pathogenesis and development of DN. Herein, we constructed a population of infiltrated immune and stromal cells and identified a new immune-related marker for DN according to the association between transcriptional levels and population abundance.

CCL19, a member of the chemokine family, has been detected in many diseases that regulate inflammation (Cait et al., 2018) and promote cell fibrosis (Bonacchi et al., 2003). Moreover, CCL19 can specifically bind to the chemokine receptor CCR7, which is expressed in semimature and mature dendritic cells (DCs), primary B cells and T cells (Comerford et al., 2013; Baran et al., 2019). Increasing evidence has revealed that CCL19 binds to CCR7 expressed on immune cells and promotes their migration and aggregation (Yan et al., 2019; Yan et al., 2021). Thus, the CCL19/CCR7 axis is a promising anti-inflammatory target in various diseases. Liu *et al.* reported that baicalin inhibits the inflammatory response in mice with ovalbumin-induced asthma *via* the CCL19/CCL21/CCR7 axis (Liu et al., 2016). In addition, CCL19 mediates diet-induced obesity and insulin resistance, and epicatechin decreases CCL19 expression in adipose tissue and inhibits diet-induced obesity and insulin resistance (Sano et al., 2017). Furthermore, previous studies found that CCL19 is upregulated in inflamed islets in nonobese diabetic mice (Shan et al., 2014) and type 2 diabetes (Santopaolo et al., 2021), suggesting that CCL19 is widely upregulated in both type 1 and 2 diabetes.

The significant function of CCL19 in the pathogenesis of human kidney diseases has also been a concern. Tertiary lymphoid organs (TLOs) are correlated with the progression of multiple chronic kidney injuries, and CCL19 is a TLO-related chemokines and highly expressed in patients with renal TLOs (Luo et al., 2021). MicroRNA-325-3p is an anti-inflammatory agent that suppresses renal inflammation and fibrosis in diabetic nephropathy by directly downregulating CCL19 (Sun et al., 2020). In addition, CCL19 also participates in the oncogenesis and progression of renal cancer. CCL19 is a critical component of an immune-related prognostic classifier for the prognostic evaluation of patients with papillary renal cell carcinoma (Wang et al., 2021). In addition, the CCL19/CCR7 axis could be a promising target for tumor gene therapy in type 2 papillary renal cell carcinoma according to Liu *et al.*’s report (Liu et al., 2020). All the evidence demonstrates that CCL19 might be a critical biomarker and therapeutic target in human kidney diseases.

In this research, we analyzed the immune-related marker transcriptome changes in the gene expression profile of 18 DN and 28 healthy tubule samples and identified 63 DEIGs, including 39 downregulated and 24 upregulated immune-related genes, in the DN samples compared to the healthy samples. Most of these upregulated genes in the DN samples were involved in the inflammatory response, regulation of T-helper 1 cell differentiation, and chemokine-mediated signaling pathways. Previous research has demonstrated that immune and inflammatory responses are among the most important factors in the pathogenesis of DN (Ram et al., 2020; Zhou et al., 2020; Shao et al., 2021). Thus, we further explored genes associated with the population abundance of tissue-infiltrated immune cells. By utilizing the MCP-counter algorithm, we estimated the abundance of infiltrated immune and stromal cell populations in the DN and healthy samples. Then, we constructed a weighted coexpression network to further investigate the association between gene expression values and the abundance of infiltrated immune cells. According to the WGCNA, DEGI analysis and PPI network, CCL19 was identified as an immune-related biomarker of DN, which was then confirmed in another independent dataset and by western blotting and a high glucose stimulation experiment, suggesting that CCL19 plays a key role in the pathogenesis of DN.

Notably, although our results indicated that fibroblasts were not different between the DN and normal samples, previous studies found that myofibroblasts that overproduce extracellular matrix are present in the renal interstitium in diabetic patients but not in normal kidneys (Ina et al., 2002; Gerrits et al., 2020), indicating that a subpopulation of fibroblasts that can be transformed into myofibroblasts play an important role in the pathogenesis and development of DN. However, because of microarray and RNA-seq limitations, it is difficult to explore the subpopulations of fibroblasts that are specifically enriched in DN patients.

## Conclusion

In summary, by performing multiple bioinformatics analyses, this study provides insights on the association between gene expression and tissue-infiltrated immune cell population abundance and identifies CCL19 as an immune-related biomarker in the pathogenesis of DN, thus providing novel evidence and a potential target for further studies on the immunologic mechanism underlying DN.

## Data Availability

The datasets presented in this study can be found in online repositories. The names of the repository/repositories and accession number(s) can be found in the article/Supplementary Material.
