# Supplementary material for: Identification of CCL19 as a Novel Immune-Related Biomarker in Diabetic Nephropathy
Source: Front Genet. 2022 Feb 9;13:830437. doi: 10.3389/fgene.2022.830437 (PMC8864156; doi:10.3389/fgene.2022.830437)
Supplement: Supplementary file 1 [file DataSheet1.docx]

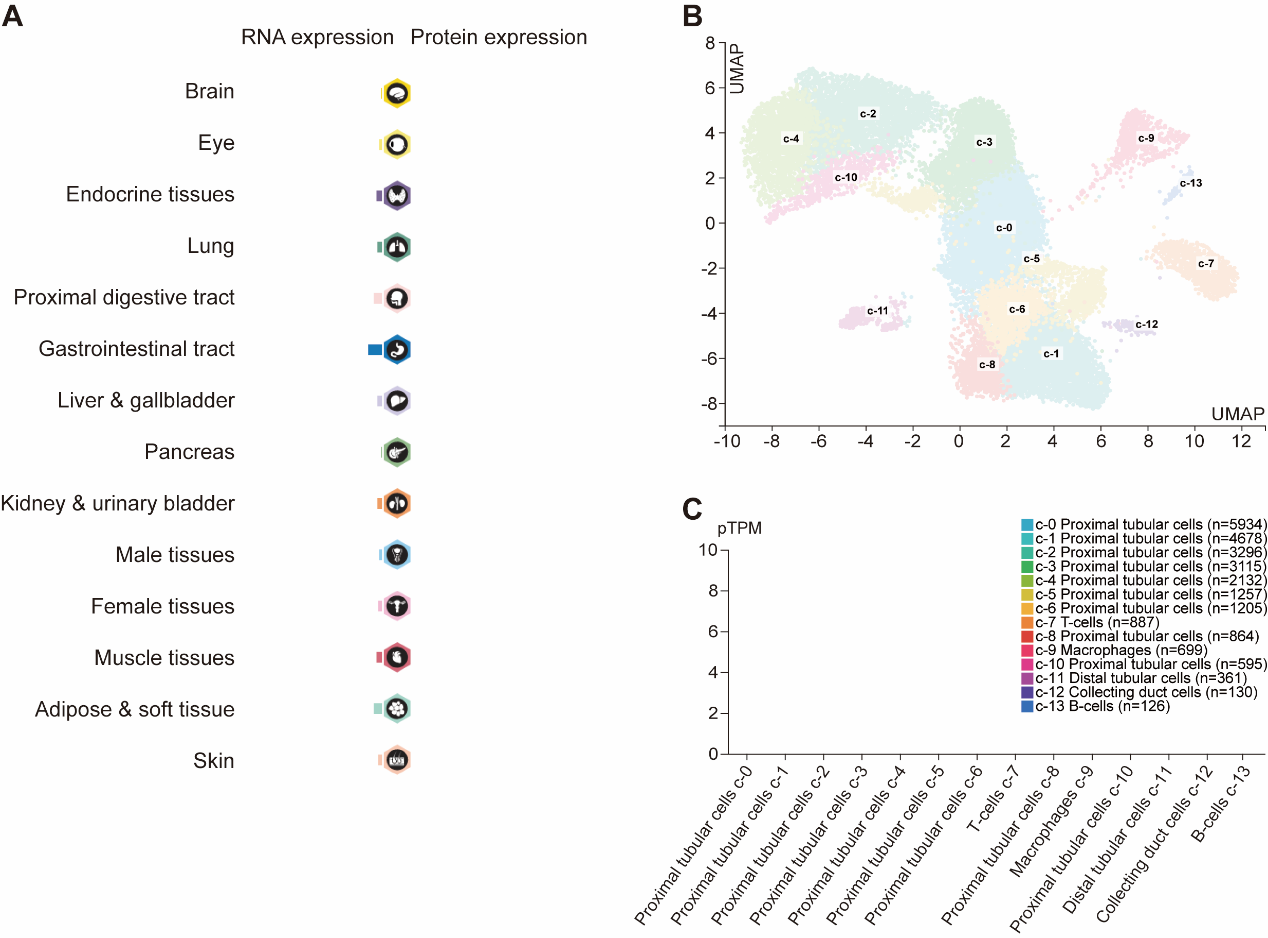


**Figure S1. The landscape of CCL19 protein in human tissues**

(A) The levels of CCL19 protein expression in human tissues. The result was acquired from the HPA dataset. (B, C) The landscape of CCL19 expression in various cell types in the kidney tissues. The result was acquired from the HPA dataset.
